# Combination of Etoposide and quercetin-loaded solid lipid nanoparticles Potentiates apoptotic effects on MDA-MB-231 breast cancer cells

**DOI:** 10.1016/j.heliyon.2024.e31925

**Published:** 2024-05-24

**Authors:** Reza Afarin, Fatemeh Ahmadpour, Mahdi Hatami, Sajad Monjezi, Somayeh Igder

**Affiliations:** a Cellular and Molecular Research Center, Medical Basic Sciences Research Institute, Ahvaz Jundishapur University of Medical Sciences, Ahvaz, Iran; b Nutritional Health Research Center, Lorestan University of Medical Sciences, Khorramabad, Iran; c Department of Clinical Biochemistry, Faculty of Medicine, Ahvaz Jundishapur University of Medical Sciences, Ahvaz, Iran

**Keywords:** Solid lipid nanoparticles (SLN), Quercetin (QC), Etoposide (ETO), MDA-MB-231, Apoptosis

## Abstract

**Background:**

Breast cancer is a major global cancer, for which radiation and chemotherapy are the main treatments. Natural remedies are being studied to reduce the side effects. Etoposide (ETO), a chemo-drug, and quercetin (QC), a phytochemical, are considered potential factors for adaptation to conventional treatments.

**Objectives:**

The anticancer effect of the synergy between ETO and Quercetin-loaded solid lipid nanoparticles (QC-SLNs), was investigated in MDA-MB-231 cells.

**Methods:**

We developed QC-SLNs for efficient cellular delivery, characterizing their morphology, particle size, and zeta potential. We assessed the cytotoxicity of QC-SLNs and ETO on breast cancer cells via the MTT assay. Effects on apoptosis intensity in MDA-MB-231 cells have been detected utilizing annexin V-FITC, PI, and caspase activities. Real-time PCR assessed Bax gene and Bcl-2 gene fold change expression, while Western blot analysis determined p53 and p21 protein levels.

**Results:**

Spherical, negatively charged QC-SLNs, when combined with ETO, significantly enhanced inhibition of MDA-MB-231 cell proliferation compared to ETO or QC-SLNs alone. The combined treatment also notably increased the apoptosis pathway. QC-SLNs + ETO increased the Bax/Bcl-2 gene ratio, elevated p53 and p21 proteins, and activated caspase 3 and 9 enzymes. These results indicate the potential for QC-SLNs + ETO as a strategy for breast cancer treatment, potentially overcoming ETO-resistant breast cancer chemoresistance.

**Conclusion:**

These results suggest that QC-SLN has the potential to have a substantial impact on the breast cancer cure by improving the efficacy of ETO. This enhancement could potentially help overcome chemoresistance observed in ETO-resistant breast cancer.

## 1 Introduction

One of the most dangerous variants of breast cancer (BC) is triple-negative breast cancer (TNBC), which is distinguished by a lack of estrogen or progesterone receptors [1]. Despite progress in cancer treatment, issues such as drug resistance, cytotoxic side effects, and metastasis persist, underscoring the need for innovative therapies [2]. Therefore, it is imperative to develop more potent strategies for treating breast cancer. These strategies should target cancer cells with abnormal cell cycle profiles or induce apoptosis. Researchers are actively searching for natural products or their analogs that can arrest cell cycle progression and trigger apoptosis without harming healthy cells [3]. Quercetin (QC), is a flavonoid belonging to the polyphenol family found in vegetables and fruits. The health implications of quercetin are intimately tied to its bioavailability, with absorption, metabolism, and *in vivo* excretion playing crucial roles in determining its overall impact [4,5]. The natural antioxidant quercetin has exhibited a multitude of pharmacological attributes in both preclinical and clinical investigations, showcasing notable anti-inflammatory and anti-cancer properties. Its capacity to regulate pivotal cell signaling pathways, such as NF-κB, p53, and STAT3, crucial in the initiation and progression of cancer, has elevated its status as an impactful anticancer agent. Considering that inflammation is a significant component in developing cancer, it is logical to conclude that quercetin, like other flavonoids such as curcumin, has strong anti-inflammatory actions. These actions can potentially alleviate inflammation and decrease the generation of reactive oxygen species (ROS) by suppressing NF-kB. Additionally, this mechanism is involved in reducing fibrosis, which is controlled by TGFβ, a process that is also regulated by NF-kB transcription [6,7]. Due to its lipophilic and hydrophobic nature, Quercetin presents difficulties in terms of its solubility in water. However, it is soluble in ethanol, dimethyl sulfoxide (DMSO), and acetone. The body's limited ability to absorb it results in significantly low levels of the substance in the bloodstream after it is taken orally. Furthermore, the stability of quercetin is dependent on the pH level, with rapid degradation occurring in alkaline settings. Quercetin is metabolized through reduction and conjugation when taken orally, resulting in the formation of metabolites that are conjugated with glucuronic acid and sulfate in the plasma. However, the biological activity of these metabolites diminishes dramatically when compared to quercetin itself. To improve its absorption, we employed solid lipid nanoparticles (SLNs) as a carrier to encapsulate quercetin in this work. The objective of this strategy is to enhance the *in vivo* distribution, availability, and stability of quercetin [8,9].

Etoposide (ETO) belongs to the class of drugs categorized as topoisomerase II inhibitors. Its mechanism of action involves the inhibition of DNA topoisomerase II, an enzyme crucial for the separation and rejoining of both DNA strands during replication. By impeding DNA re-ligation, ETO induces errors in DNA synthesis and cell division. Additionally, ETO induces DNA damage by stabilizing a topoisomerase II and DNA complex that is cleavable. Its primary activity occurs during the late S and G2 phases of the cell cycle [10,11]. However, due to its drug resistance, accompanied by high dosages, and negative side effects, using ETO as a sole agent for BC treatment has been linked to low response and recurrence rates of cancer [12]. Etoposide is often used in combination with other drugs to treat malignant disorders [[13], [14], [15]]. The natural remedy QC has been shown to increase the effectiveness of ETO against cancer. Combining QC with chemotherapeutic agents can enhance the efficiency of conventional chemotherapy [[16], [17], [18]]. This paper aims to assess the synergistic effects of ETO and QC-loaded SLNs on the breast cancer cell line MDA-MB-231. We are examining key factors associated with the apoptosis pathway. Furthermore, the impact of QC-loaded SLNs on the effectiveness of low-dose ETO has not been previously investigated.

## 2 Materials and methods

### 2.1 Synthesis of QC- SLNs

100 mg QC was mixed with glyceryl Dibehenate (Campritol 888 ATO, France) at 75 °C. Simultaneously, oleic acid (0.25 g) and lecithin (0.5 g) were blended with deionized water at 80 °C for 5 min before being combined with the primary solution using an ultrasonic device (Elmasonic S60H, Global Industrial, USA). The previous mixture was mixed with 1 % polyvinyl alcohol (PVA) (4 ml) and homogenized at 10,000 rpm with a homogenizer (Heidolph, Germany) to create the nanoemulsion. Centrifuging was done twice for 25 min at 5 °C and 15,000 rpm on the suspension that resulted. The resulting QC-SLNs were then kept at cold temperatures in closed containers until needed.

### 2.2 Fourier transform infrared (FTIR) spectroscopy

To confirm intermolecular interactions following the incorporation of QC into SLNs, we analyzed FTIR spectra using a VERTEX 70v instrument from Bruker of the USA. Pellets formed by compressing QC, QC-SLN, and blank-SLN with KBr. FTIR spectra of materials were recorded at 400-4000 cm⁻^1^ resolution, with 1 cm⁻^1^ resolution.

### 2.3 Transmission Electron Microscopy (TEM) analysis

SLN morphology was examined using TEM. A droplet of the SLN was used to cover a copper grid that had been coated with carbon, forming a thin liquid layer. Extra samples were gathered on filter paper and allowed to air dry. A TEM device (ZEISS LEO 906 E) was then used to analyze the SLN morphology.

### 2.4 Particle size, zeta potential, and PDI measurements

To determine the average particle size, zeta potential, and polydispersity index (PDI) of QC-SLNs, a nanosizer and zetasizer from Malvern, England, were employed.

### 2.5 Encapsulation efficiency (EE) and drug loading (DL) assessment

To estimate the encapsulation efficiency (EE) initially, free (untrapped) QC was separated from QC-SLNs in the suspension. Centrifugation was used to achieve this separation, which was conducted for approximately 20 min at 25,000 rpm. Using a UV spectrophotometer, the supernatant's QC concentration was measured at 256 nm. EE (%) = 100 × (Di - Df)/Di, where Di and Df represent the initial (total) and free (untrapped) drug concentrations, respectively [19,20].

QC-SLN was also dissolved in methanol to measure the QC content, and the drug loading (DL) was calculated as follows: DL (%) = 100 × (loaded drug/weight of lipid).

### 2.6 In vitro drug release

Dialysis bags (Sigma-Aldrich, USA) with a molecular weight limit of 12,000 Da were used in the experiment [21]. QC release from QC-SLNs was assessed in phosphate-buffered saline (PBS) at a pH of 7.42, serving as the receptor phase. Experiments were carried out at 37 °C. Samples were stored for predetermined times (10 min–72 h). The QC content of the samples was subsequently determined using spectrophotometry, specifically the Ultrospec 3000 from Pharmacia Biotech in the USA, at a wavelength of 256 nm.

### 2.7 Cell culture and experimental design

MDA-MB 231 cells were obtained from the Pasteur Institute collection of cell cultures (Tehran, Iran) and were cultured in Dulbecco's Modified Eagle Medium (DMEM) high glucose medium with 1 % penicillin/streptomycin, 10 % fetal bovine serum (FBS), and penicillin. They were obtained from the Pasteur Institute of Iran and incubated at 37 °C, 95 % humidity, and 5 % CO2.

### 2.8 Cell viability assessment

The study utilized the MTT assay to evaluate the impact of treatments on cell viability. Separate 96-well plates were used to seed cells (4 × 10³ cells/well), and they were left to incubate overnight. Fresh DMEM containing specific doses of ETO (10–100 μM), and QC-SLN (5–80 μM), either alone, was used to replace the media. New media containing 10 % MTT was added to the wells after the media was removed following 24, and 48 h of incubation. The media containing MTT were substituted with dimethyl sulfoxide (DMSO) solutions to dissolve the formazan crystals after 4 h. The absorbance was established at 570 nm via a Microplate reader from BioTek, USA. The percentage of cell viability in comparison to the control cells was given. The IC50 values were estimated using GraphPad Prism software (version 8.0).

### 2.9 Determining combination index (CI) and Dose Reduction Index (DRI)

To assess the synergistic relationship between ETO and QC-SLN, we calculated CI and DRI through CompuSyn software (Chou and Martin, 2005, CompuSyn Inc, USA). CI values were used to evaluate the combined effects. A CI value of 1 indicates additive effects, while values greater than 1 suggest antagonism and values less than 1 indicate synergy. Additionally, DRI was employed to quantify the correlation between drug dosages and the reduction in drug dosage. A DRI value greater than 1 is considered favorable and implies drug synergy.

### 2.10 Flow cytometry assessment

The cells' apoptotic condition was evaluated through flow cytometry by utilizing the Annexin V-fluorescein isothiocyanate (FITC)/Propidium iodide (PI) kit (IQ Products, Groningen, Netherlands) under the guidelines provided by the manufacturer. After treatment with ETO and QC-SLN, a specific number of suspended and trypsinized cells were collected, washed with calcium buffer, and centrifuged to obtain a cell pellet. Cells were then resuspended in the same buffer supplemented with Annexin V-FITC and incubated at 4 °C for 20 min. The buffer was subsequently replaced with calcium buffer containing PI, and the cells were kept in an incubator for an extra 10 min at the same temperature. Finally, the flow cytometer (Becton, Dickinson, San Jose, CA, USA) was used to examine the samples. Apoptotic status was determined by analyzing Annexin V-FITC/PI staining patterns. FlowJo software version 10 was utilized for data analysis.

### 2.11 Caspase-3 and 9 activity assay

Caspase-3 and Caspase-9 activity within the cell lysate samples was measured using the Caspase 3 Assay Kit, Colorimetric (Abcam; ab39401, USA). Cells were collected, resuspended in lysis buffer, and centrifuged to obtain cell extracts. The determination of protein content was carried out for every sample and specific volumes of cell extract containing equivalent amounts of protein were collected. The activities of Caspase-3 and Caspase-9 were evaluated by incubating samples with enzyme-specific colorimetric substrates for 1 h, followed by measuring absorbance at 405 nm to evaluate substrate cleavage.

### 2.12 Real-time PCR analysis

Once the cellular density reached 70–80 %, the cells underwent treatment with ETO and QC-SLN for 48 h. RNA extraction was conducted following the manufacturer's guidelines, with RNX-Plus Solution, with a total of 1 × 10^6^ cells. To assess RNA purity and integrity, two methods were employed: Agarose gel electrophoresis and A260/A280 ratio. Isolated RNA was stored at −70 °C in 50 μl of DEPC-treated water. A cDNA synthesis kit was used for reverse transcription under the manufacturer's guidance in a 20 μl reaction mixture. The expression of Bax, Bcl-2, and GAPDH genes was quantified using SYBR Green qPCR Master Mix from Yekta Tajhiz Azuma in Tehran, Iran, along with specific primers. Specific primer pairs were freshly designed for each gene, and the sequences are in Table 2. At the end of PCR cycles, a melt curve plot was generated to prove the product uniformity. The PCR steps are: 1. Initial denaturation: The reaction was incubated at 96 °C for 10 min 2. Cycling: The reaction underwent 40 cycles, each consisting of the following steps: a. Denaturation: The reaction was heated to 95 °C for 15 s. b. Annealing: The reaction was cooled to 60 °C for 30 s. c. Extension: The reaction was incubated at 60 °C for 34 s. The function of GAPDH was to serve as a housekeeping gene to normalize expression levels. The 2^^(−ΔΔCT)^ approach was used to calculate fold changes. The sequence of primers utilized in this study is presented in Table 1.

**Table 1:** Primers related to BAX, Bcl2, Caspase-3 and GAPDH.

Name	Forward	Reverse
Bax	5′-CAGGGGCCCTTTTGCTTCA-3′	5′-ACGGCGGCAATCATCCTCT-3′
Bcl2	5′-GGATAACGGAGGCTGGGATG-3′	5′-TGACTTCACTTGTGGCCCAG-3′
Caspase-3	5′-AGGACTCTAGACGGCATCCA-3′	5′-CAGTGAGACTTGGTGCACTGA-3′
GAPDH	5′-ACCCTTAAGAGGGATGCTGC-3′	5′-CCCAATACGGCCAAATCCGT-3′

**Table 2:** Combination Index (CI) and Dose Reduction Index (DRI) between QC-SLN and Etoposide in MDA-MB-231 cells.

	QC-SLN (5 μM)	QC-SLN (10 μM)	QC-SLN (20 μM)
CI (Etoposide 10 μM)	0.89	0.73	0.49
DRI	8	4	2

**Note:** MDA-MB-231 cells were incubated with 10 nM of Etoposide and various concentrations of QC-SLN for 48h. CI was calculated by CompuSyn software. CI < 1.0 represents synergism, CI = 1.0 represents an additive, and CI > 1.0 represents antagonism. DRI (>1) means that the dose of each drug in the combination can be reduced to achieve the same effect level compared to the doses of each drug alone, which can potentially reduce side effects.

### 2.13 Western blot analysis

After the cells reached a density of 70–80 %. Then they were treated with ETO and QC-SLN for 48 h. Breast cancer cells subjected to treatment were initially washed with cold PBS (pH 7.42). Following this, they were collected in a RIPA lysis buffer containing protease inhibitors. After conducting SDS-PAGE and blotting, a PVDF membrane was blocked using a 5 % non-fat milk solution. The membranes were treated with primary antibody against p53 (1: 1000; sc-47698), p21 (1: 1000; sc-271610) and GAPDH (1: 1000; sc-32233), proteins (Santa Cruz Biotechnology Inc., USA). After three washes with PBS‐Tween20, Peroxidase-conjugated goat anti-rabbit IgG secondary antibody (1: 10,000; Santa Cruz Biotechnology Inc., USA) was incubated with the membranes at room temperature for 1 h protein bands were visualized using the ECL kit from Abcam, USA. Band density quantification was carried out using Image J software, developed by the National Institutes of Health in Bethesda, United States.

### 2.14 Statistical analysis

Version 8.0 of GraphPad Prism software was used for data analysis. Results are shown as mean standard deviation (SD). One-way analysis of variance (ANOVA), followed by Tukey's multiple comparison tests, was used to establish the statistical significance. Statistics were considered significant for p-values under 0.05.

## 3 Results

### 3.1 Characterization and drug release of QC-SLNs

**Fig. 1:**
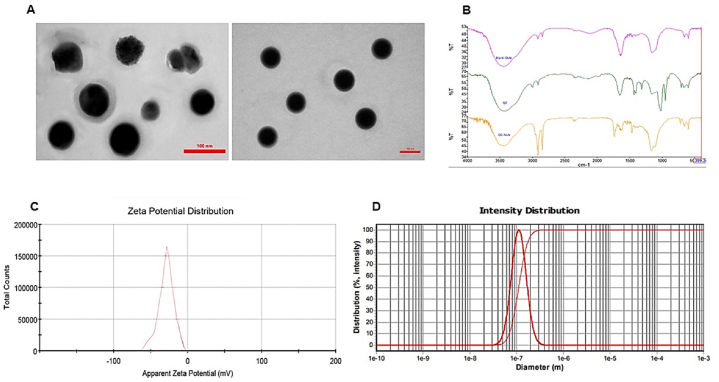
(A) a Transmission Electron Microscopy (TEM) micrograph of Quercetin -loaded solid lipid nanoparticles (QC-SLNs). Scale bar: 100 nm; (B) The FTIR spectra for QC-SLNs. (C) The ζ-potential for QC-SLNs. (D) The average size distribution for QC-SLNs.

The production of QC-SLN using the low-temperature emulsification and solidification methods has been previously documented [22]. Initially, TEM was employed to reveal the size and morphology of these particles. QC-SLNs were found to be smooth, solid, spherical particles with a narrow polydispersity index (PDI = 0.50 ± 0.04), as depicted in Fig. 1. The mean particle size of SLNs was observed to be around 104 ± 12.5 nm, consistent with the dynamic light scattering (DLS) measurement of 112 nm. Notably, the average particle size fell below the expected range of 112 nm.

Two critical factors influencing how nanoparticles affect cancer cells are their surface charge and size. The ζ-potential plays a pivotal role in maintaining nanoparticle stability in suspension due to electrostatic repulsion between particles. The ζ-potential value of QC-SLN was approximately −25.8 mV, which is sufficiently high to deter nanoparticle aggregation, ensuring long-term stability in the suspension. Fig. 1 illustrates the distinct release profiles of QC solution, with QC-SLN demonstrating sustained release over three days. It released 65 % of the drug within the first 12 h, gradually increasing to 90 % over subsequent days.

### 3.2 FTIR analysis

FTIR spectrum featuring distinct band peaks. About quercetin structure, the absorption peak at (3820–3150 cm-1), could correspond to O–H stretching, 1625 cm-1 was related to C=O stretching, 1670 cm-1 assigned to C–C stretching, (1455, 1385 cm-1) linked to C–H bending, 1257 cm-1 assigned to C–O stretching in the ring structure, and the absorption at (1115 -1062 cm-1) was related to C–O stretching in the ring structure. Markedly, no deletions were found in the peaks of the QC-SLN functional group, proving that the formulation of QC-SLN used the QC's corresponding structure in conjunction with other components (Fig. 1A–D).

### 3.3 Dose Reduction Index (DRI) measurement

In our study, we calculated the DRI for the combination of 10 μM Etoposide (ETO) and 20 μM of Quercetin-loaded Solid Lipid Nanoparticles (QC-SLNs) using the formula:

$$DRI=\frac{{IC}_{50}\;of\;drug\;alone}{{IC}_{50}\;of\;drug\;in\;combination}$$

For the combination of 10 μM ETO and 20 μM QC-SLNs, we found:

$${DRI}_{20QC-SLN}=\frac{{IC}_{50}\;\text{of}\;\text{QC}-\text{SLN}\;\text{alone}}{{IC}_{50}\;\text{of}\;\text{combination}\;\text{of}\;\text{ETO}\;\text{and}\;20\;\mu M\;\text{QC}-\text{SLNs}}=\frac{40\mu M}{20\mu M}=2$$

This DRI value of 2 suggests that the dose of QC-SLN could potentially be reduced by half when used in combination with ETO, compared to when used alone, to achieve the same effect. This is a significant finding as it indicates a synergistic effect between ETO and QC-SLNs, allowing for dose reduction and potentially reducing side effects. Furthermore, among the combinations tested, the one with the lowest combination index (0.49), which included the highest QC-SLN concentration (20 μM) and the lowest ETO concentration (10 μM), was selected for further experiments. This combination not only showed a synergistic effect but also allowed for a significant reduction in the ETO dose, further emphasizing the potential benefits of this drug combination.

### 3.4 QC-SLNs and ETO act synergistically on the inhibition of BC cell growth

To determine the optimal concentrations for QC-SLN and ETO in inhibiting MDA-MB231 cell growth, MTT assays were conducted. Different concentrations ranging from 10 to 100 μM for ETO and 5–80 μM for QC-SLN were used at both 24 and 48 h. The results showed that both QC-SLN and ETO exhibited dose- and time-dependent effects on cell growth.

**Fig. 2:**
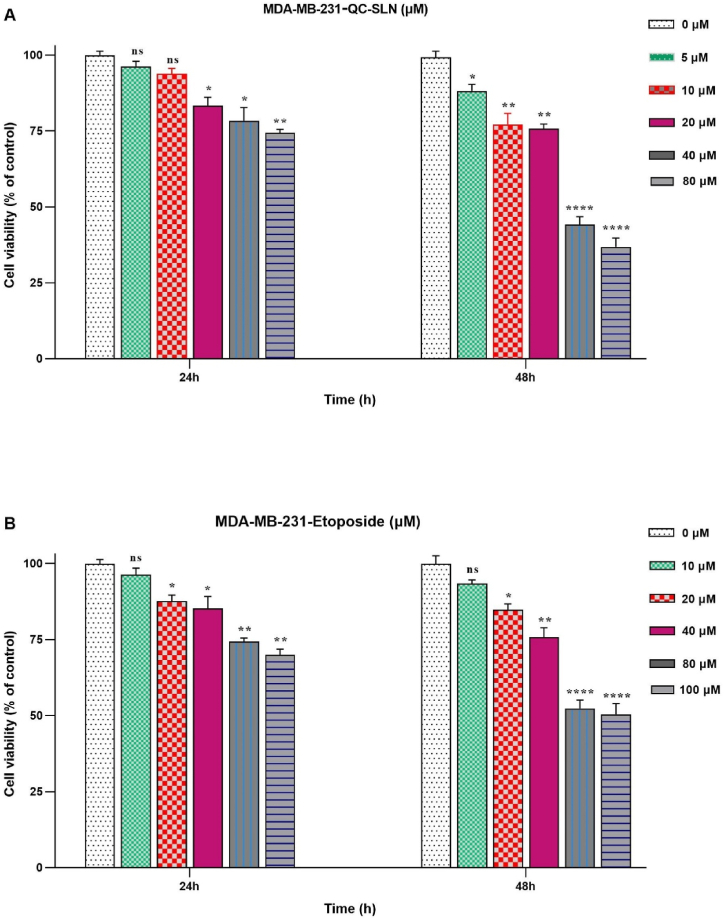
Cell Viability determination through MTT Assay in MDA-MB-231 cells. Cells were treated with various concentrations of Etoposide (ETO) and Quercetin-loaded solid lipid nanoparticles (QC-SLNs), and incubated for 24 h and 48 h. ns: not significant, *p < 0.05 or **p < 0.01 or ***p < 0.001, ****p < 0.0001 as compared to control (mean ± SD). The results have been analyzed after three repetitions.

After 24 h, there was little variation in cell growth among the five different drug concentrations. However, after 48 h, it became evident that doses of 80 and 100 μM ETO significantly reduced tumor cell growth. Similarly, the highest inhibitory effects of QC-SLN on tumor cells were observed at doses of 40 and 80 μM (Fig. 2).

The IC50 values were calculated for ETO and QC-SLN, and these values were subsequently used in the combination experiments. The decision was made to combine QC-SLN concentrations of 5, 10, and 20 μM with an ETO concentration of 10 μM (Fig. 2A and B).

**Fig. 3:**
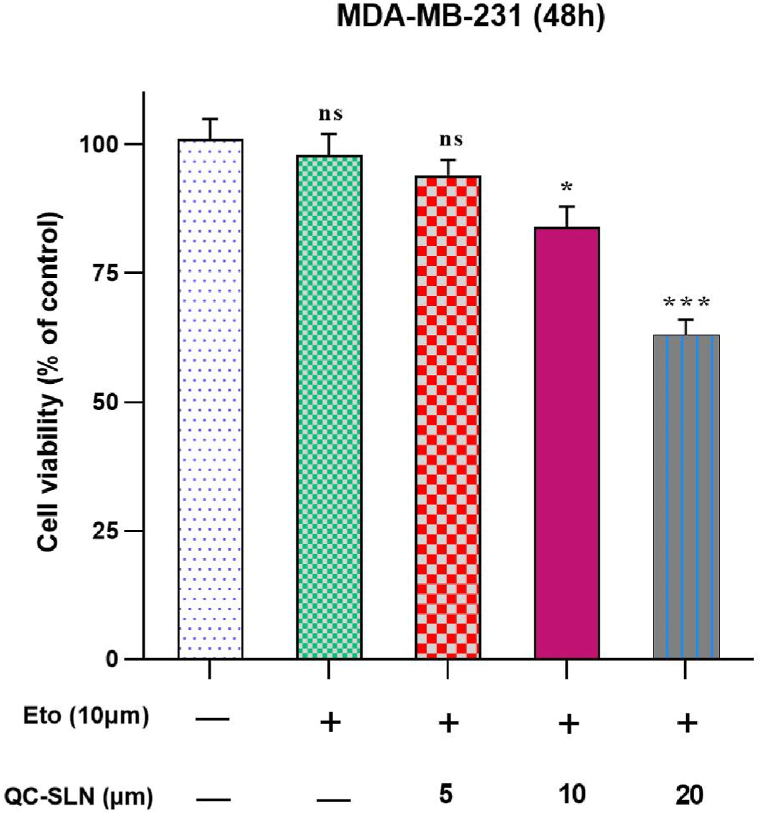
Cell Viability determination through MTT Assay in MDA-MB-231 cells. Cells were treated with one concentration of Etoposide (ETO) with various concentrations of Quercetin -loaded solid lipid nanoparticles (QC-SLNs), for 48 h; (10 μM of ETO combined with 5, 10, and 20 μM of QC-SLNs). ns: not significant, *p < 0.05 or **p < 0.01 or ***p < 0.001, as compared to control (mean ± SD). The results have been analyzed after three repetitions.

According to the findings, ETO demonstrated inhibitory activity at a concentration of 10 μM when combined with 10 and 20 μM QC-SLNs, with only minor differences in cell viability compared to higher concentrations of ETO alone (Fig. 3). The combination indices indicated that QC-SLNs and ETO worked synergistically. Among the combinations tested, the one with the lowest combination index (0.49), and Dose Reduction Index (DRI) greater than 1, which included the highest QC-SLN concentration (20 μM) and the lowest ETO concentration (10 μM), was selected for further experiments (Table 2).

### 3.5 Effect of ETO + QC-SLNs combination on protein levels of P53 and P21 and Bax and Bcl-2 gene expression

**Fig. 4:**
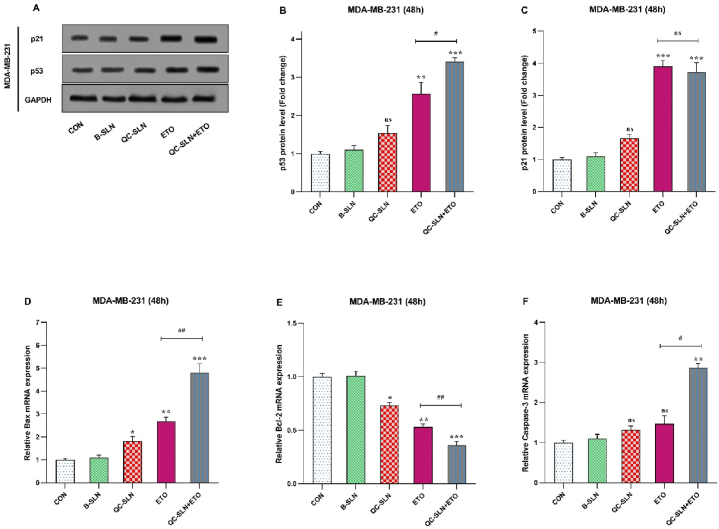
Effects of Quercetin-loaded solid lipid nanoparticles (QC-SLNs) and Etoposide (ETO) on the relative expression of apoptosis-related protein genes in MDA-MB-231 cells; A, Western blot gel image of p53 and p21 proteins; B, P53 protein levels in MDA-MB-231 cells treated with QC-SLNs and ETO alone and in combination; C, P21 protein levels in MDA-MB-231 cells treated with QC-SLNs and ETO alone and in combination. D, BAX gene expression in MDA-MB-231 cells treated with QC-SLNs and ETO alone and in combination; E, Bcl-2 relative gene expression in MDA-MB-231 cells treated with QC-SLNs and ETO alone and in combination; F, Caspase 3 relative gene expression in MDA-MB-231 cells treated with QC-SLNs and ETO alone and in combination (ns: not significant, *p < 0.05 or **p < 0.01 or ***p < 0.001, ****p < 0.0001 as compared to control, #p < 0.05, ##p < 0.01 compared to ETO alone) (mean ± SD). The results have been analyzed after three repetitions.

To further investigate the induction of apoptosis in MDA-MB-231 cells, the levels of the p53 and p21 proteins were measured (Fig. 4. A, B, C). The results indicated that exposure to ETO alone led to a significant increase in the levels of the tumor suppressor proteins p53 and p21 in the tested cells. Notably, QC-SLN enhanced the impact of ETO on p53 and p21 proteins, as demonstrated in this study. A 2.57-fold increase in p53 levels was caused by the ETO treatment, while QC-SLN improved its impact, with a 3.42-fold increase. ETO treatment also increased p21 levels 3.9-fold, although, QC-SLN treatment reduced its effect by a 3.72-fold decrease. (QC-SLN treatment decreased the effect of ETO treatment by 3.72 times, while p21 levels increased by 3.9 times).

The combination of QC-SLN + ETO was found to modulate the expression of Bcl-2 and Bax genes, promoting apoptotic cell death. According to the study's findings, QC-SLN and ETO individually increased the expression of the Bax gene by 1.8 and 2.7-fold, respectively. However, when these two substances were combined, an additive effect on the Bax gene, increased its expression up to 4.8-fold, as depicted in Fig. 4. Conversely, exposure to the QC-SLN and ETO combination led to a decrease in Bcl-2 expression to 0.36-fold. In contrast, Bcl-2 expression decreased to 0.73- and 0.52-fold after treatment with QC-SLN and ETO, respectively (Fig. 4. D, E, F).

### 3.6 Potential effect of ETO + QC-SLNs combination on apoptosis flow cytometry in BC cells

**Fig. 5:**
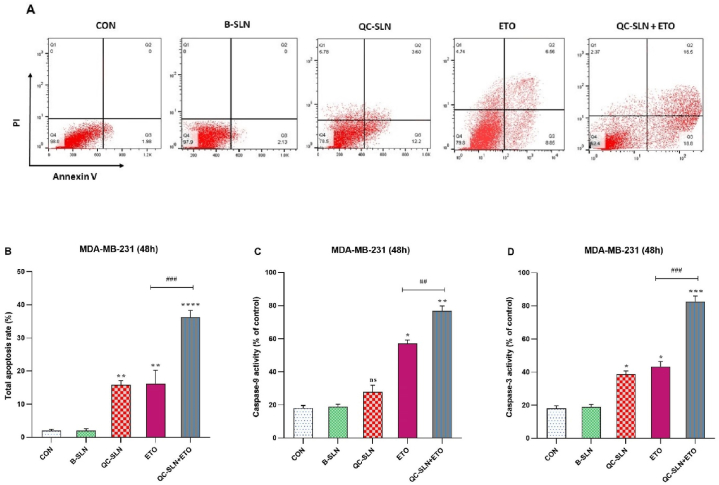
Effects of Quercetin -loaded solid lipid nanoparticles (QC-SLNs) and Etoposide (ETO) on the apoptosis of MDA-MB-231 cells through flow cytometry; A, flow cytometry plot of the effects of QC-SLNs and ETO individually and in combination on the MDA-MB-231 cell death; B, columnar plot of flow cytometry for QC-SLNs and ETO; C, Caspase-9 activity in MDA-MB-231 cells treated with QC-SLNs and ETO alone and in combination; D, Caspase-3 activity in MDA-MB-231 cells treated with QC-SLNs and ETO alone and in combination (ns: not significant, *p < 0.05 or **p < 0.01 or ***p < 0.001, ****p < 0.0001 compared to control; #p < 0.05, ##p < 0.01, ###p < 0.0001 compared to ETO alone) (mean ± SD). The results have been analyzed after three repetitions.

Annexin/PI staining, a technique that combines two dyes with unique staining characteristics, was utilized to investigate the induction of apoptotic subpopulations in cells. In contrast to untreated cells, we provide evidence that both QC-SLN and ETO substantially enhance programmed cell death. The efficacy of QC-SLNs is enhanced to 16.2 % ETO and 15.8 % apoptosis. The interaction between the activities of the two ingredients is further illustrated in Fig. 5. A, where the combined effects of QC-SLN and ETO on cells led to a 36.2 % increase in the rate of programmed cell death (supplementary materials).

### 3.7 *Potential effect of ETO + QC-SLNs combination on* caspase-9 and caspase-3 activity *in BC cells*

The analysis of the activities of caspase-9 and caspase-3 in MDA-MB-231 cells comprised the following section. In the absence of ETO and QC-SLN, caspase-9 activity was reduced by 28 % and 57 %, respectively. A 76.5 % increase in caspase-9 activity was observed with the combination of QC-SLN and ETO. In addition, the activity of caspase-3 was increased by 39 % and 43 %, respectively, when treated exclusively with QC-SLN and ETO. The combination of QC-SLN and ETO significantly increased the activity of caspase-3, which reached 82 % (Fig. 5. B, C, and D).

## 4 Discussion

The development of effective anticancer drugs faces challenges due to low therapeutic indices, drug resistance, and tumor accumulation. Access to healthcare and improved diagnostic equipment is crucial for reducing breast cancer incidence [23]. Nanotechnology, combining phytochemicals with nanoparticles, is being explored for targeted drug delivery, enhancing therapeutic benefits and minimizing side effects [24]. Studies have indicated that quercetin (QC) reduces cell viability and triggers apoptosis through intrinsic and extrinsic caspase-dependent pathways in MDA-MB-231 cells. This process is accompanied by heightened cytosolic Ca2+ levels, diminished mitochondrial membrane potential, activation of caspase-3, −8, and −9, upregulation of FAS and Bax, and downregulation of Bcl-2 and XIAP [25]. Additionally, QC induces cell cycle arrest by down-regulating cyclin A and B and up-regulating p57. Moreover, QC inhibits the expression of Hsp27, Hsp70, and Hsp90, which are implicated in cell survival in MDA-MB-231 cells. This inhibition leads to apoptosis, characterized by increased caspase activity and PARP cleavage [26,27]. Previous research has demonstrated the involvement of quercetin (QC) in modulating these proteins, leading to cell cycle arrest and apoptosis induction [23,24]. Our findings are consistent with these studies, showing that exposure to QC-SLNs alone or in combination with ETO resulted in up-regulation of p21 and p53 expression, indicating potential cell cycle arrest and tumor suppressor activity. Apoptosis, or programmed cell death, plays a crucial role in cancer prevention and therapy. Studies have reported the ability of QC to induce apoptosis through intrinsic and extrinsic pathways, involving caspase activation and modulation of Bcl-2 family proteins [23,25]. In our study, we observed enhanced apoptotic effects when QC-SLNs were combined with ETO, as evidenced by increased caspase activity and altered expression of Bax and Bcl-2. Despite research and resources dedicated to understanding cancer mechanisms, low bioavailability poses a quality control problem [28]. The current study indicates that the use of Solid Lipid Nanoparticles enhances the bioavailability of QC [29]. To determine the anticancer effectiveness of QC-SLNs on ETO breast cancer cell proliferation, we assessed the inhibitory impact of the two drugs on MDA-MB-231 cell growth in comparison to the effects of the drugs alone. We looked at the molecular mechanisms underlying cancer progression and assessed whether QC-SLNs exhibit any synergy with the chemotherapy ETO on cell viability, cell apoptosis, and caspase activation. The apoptotic process is thereby blocked as a result of the therapy.

Incorporating QC into SLNs through micro emulsification, using lipids and surfactants with stearic acid as the negatively charged solid lipid, has effectively enhanced water solubility and minimized pre-systemic metabolism. The uniform size and negative zeta potential contribute to stability. These SLNs enable controlled release of QC, making them suitable for delayed drug delivery in breast cancer chemotherapy. In QC-encapsulated SLNs, FTIR analysis revealed no drug-lipid interactions, suggesting that there were no potential interactions that would compromise the potency and stability of the encapsulation [22].

This study investigated the anti-proliferative responses of QC-SLNs synergy with the ETO to MDA-MB-231 cells. At the first, we showed time- and dose-dependent cytotoxicity manner of QC-SLNs and ETO alone. Previous research showed that QC-SLNs significantly increase the study-demonstrated cytotoxicity against both MCF-7 and MDA-MB-231 cells, with a higher accumulation of QC in loaded SLNs compared to free drugs [22]. This enhanced anticancer effect could be attributed to the transporter's lipophilic nature, which facilitates drug delivery and intracellular uptake. Other studies found that QC has an IC50 of 50 μM for inhibiting breast cancer cell proliferation. Notably, the lower IC50 of QC-SLNs (25 μM) compared to free QC suggests that QC-loaded nanoparticles exhibit increased toxicity to MCF-7 cells [30].

Importantly, there have been no prior investigations on MDA-MB-231 cells to assess the cytotoxic effects of QC-SLN in combination with ETO. The findings demonstrated that the cytotoxic effect of ETO was enhanced at the lowest concentration (10 μM) when it was combined with QC-SLNs at concentrations of 10 μM and 20 μM. This suggests that QC-SLNs may enhance the antiproliferative potency of ETO on MDA-MB-231. The combination of QC-SLN and ETO is proposed as a potential novel strategy for breast cancer treatment.

Apoptosis is a vital cell death pathway that is essential for cancer prevention. Several key targets have been identified to induce cell cycle detention, enhance cellular senescence, and ultimately lead to cell death [31]. These targets regulate important cellular factors and processes, for instance, Bcl-2, Bax, and cyclin-dependent kinases [32]. Among these, Bcl-2, known for its ability to suppress apoptosis, serves as a convergence point for multiple oncology-related signaling pathways [33].

In our study, the rates of apoptotic cell death were assessed when QC-SLNs and ETO were administered separately and in combination, with a focus on their potential involvement in the signaling pathway. The findings revealed that QC-SLNs not only promote cancer cell apoptosis but also synergistically enhance the apoptotic effect of ETO on cancer cells. The study examined the mRNA expression levels of two genes, Bax and Bcl-2, as well as two proteins, p53 and p21, and the activities of caspase-3 and caspase-9 in response to QC-SLN + ETO exposure in MDA-MB-231 cells, as these pathways are key regulators of apoptosis.

Our results demonstrated that combination treatment with QC-SLNs and ETO up-regulated the mRNA levels of the pro-apoptotic Bax and cell cycle checkpoint proteins p53 and p21, while down-regulating the expression of the anti-apoptotic Bcl-2 in treated cells. Furthermore, both apoptotic caspase-3 and caspase-9 activities were higher in cells exposed to QC-SLNs + ETO compared to individual drug treatments.

Activation of p53 triggers the activation of the Bcl-2 family proteins, including Bax. This leads to the opening of the mitochondrial outer membrane pores and the entry of soluble proteins from the membrane into the cytosol, where they activate caspases [34]. Therefore, QC-SLNs can trigger intrinsic apoptosis and induce cell damage in MDA-MB-231. The Bax/Bcl-2 ratio balance can influence the sensitivity of tumor cells to chemotherapy or radiation [35]. Previous discoveries have also stated that Bax is involved in QC-induced apoptosis in prostate cancer cells [36], and QC can activate apoptosis and decrease cell growth in MCF-7 cells by regulating the expression of Bax and Bcl-2 [37,38]. Our flow cytometry results demonstrate that MDA-MB-231 cells co-treated with QC-SLN + ETO exhibited a significant increase in the rate of apoptosis, indicating that QC-SLN can activate cell death pathways with lower levels of necrosis. This suggests that the combination of QC-SLN and ETO has a coactive outcome in promoting apoptosis in these cells.

Notably, previous research has also supported the ability of QC to induce apoptosis and necrosis in various cancer cell types [39]. For instance, a study demonstrated that QC induced apoptosis in squamous cells, aligning with the findings in this study [40]. Similarly, one study reported that QC nanoemulsifying induced DNA damage and apoptosis in MCF-7 cells [41], consistent with the current results. Additionally, some studies suggested that QC induces apoptosis by arresting the cell cycle at the G2/M and G0/G1 phases in colorectal cancer cells [42]. This aligns with the findings indicating that QC-SLN can enhance the apoptotic effects of ETO in MDA-MB-231 cells. Moreover, previous research demonstrated that QC can modulate the PI3K/Akt and NF-kB pathways either independently or with chemotherapeutic agents, ultimately inducing the mitochondrial pathway of apoptosis [43]. These results offer further support for the potential of QC-SLN in promoting apoptosis in cancer cells, particularly when combined with other therapeutic agents.

## 5 Conclusion

The present study has meticulously demonstrated that QC-SLNs exhibit a significant increase in cytotoxicity and apoptotic induction in MDA-MB-231 breast cancer cells, especially when used in conjunction with ETO. These findings extend the understanding of QC's therapeutic potential, as encapsulation in SLNs enhances its bioavailability and synergizes its anticancer effects. Through a time- and dose-dependent response, QC-SLNs have shown to not only induce apoptosis by modulating the expression of critical apoptotic and cell cycle regulatory genes and proteins, such as Bax, Bcl-2, p53, and p21 but also to augment the activity of caspases −3 and −9, crucial for the execution phase of apoptosis. The evidence from this study suggests a potential therapeutic strategy that leverages the combination of QC-SLNs with ETO, enhancing the pro-apoptotic balance in favor of Bax over Bcl-2, thereby promoting cell death and offering a promising avenue for effective breast cancer treatment. This novel approach, focusing on the intrinsic pathway of apoptosis, could potentially overcome the limitations posed by drug resistance and low therapeutic indices, thus representing a significant step forward in oncological therapeutics.

## Data availability

The authors do not have permission to share data.

## Ethical approval

The present study was designed and conducted with the permission of the Ethics Committee of Ahwaz Jundishapur University of Medical Sciences (IR.AJUMS.REC.1401.322).

## CRediT authorship contribution statement

**Reza Afarin:** Writing – original draft, Software. **Fatemeh Ahmadpour:** Writing – review & editing, Validation, Resources. **Mahdi Hatami:** Methodology, Investigation, Formal analysis. **Sajad Monjezi:** Visualization, Data curation. **Somayeh Igder:** Visualization, Supervision, Project administration, Funding acquisition, Conceptualization.

## Declaration of competing interest

The authors declare the following financial interests/personal relationships which may be considered as potential competing interests:Somayeh Igder reports a relationship with Ahvaz Jondishapour University of Medical Sciences that includes: employment. Somayeh Igder has patent pending to CMRC-0119. RA, SI designed and supervised the project. RA performed all assays in collaboration with MH. SM analyzed the data and interpreting the results. FA, and RA wrote the first draft and revised the manuscript. The authors read and approved the final manuscript. If there are other authors, they declare that they have no known competing financial interests or personal relationships that could have appeared to influence the work reported in this paper.

## Abbreviation

Etoposide: (ETO)
Quercetin: (QC)
Quercetin -loaded solid lipid nanoparticles: (QC-SLNs)
Solid lipid nanoparticles: (SLN)
Breast cancer: (BC)
Triple negative breast cancer: (TNBC)
Fourier Transform Infrared: (FTIR)
Transmission Electron Microscopy: (TEM)
Polydispersity index: (PDI)
Encapsulation Efficiency: (EE)
Drug Loading: (DL)
phosphate-buffered saline: (PBS)
Dulbecco's Modified Eagle Medium: (DMEM)
fetal bovine serum: (FBS)
Combination Index: (CI)
Dose Reduction Index: (DRI)
dimethyl sulfoxide: (DMSO)
standard deviation: (SD)
One-way analysis of variance: (ANOVA)
