# Supplementary figures and images for: Combination of Etoposide and quercetin-loaded solid lipid nanoparticles Potentiates apoptotic effects on MDA-MB-231 breast cancer cells
Source: Heliyon. 2024 May 24;10(11):e31925. doi: 10.1016/j.heliyon.2024.e31925 (PMC11152947; doi:10.1016/j.heliyon.2024.e31925)

**P53**


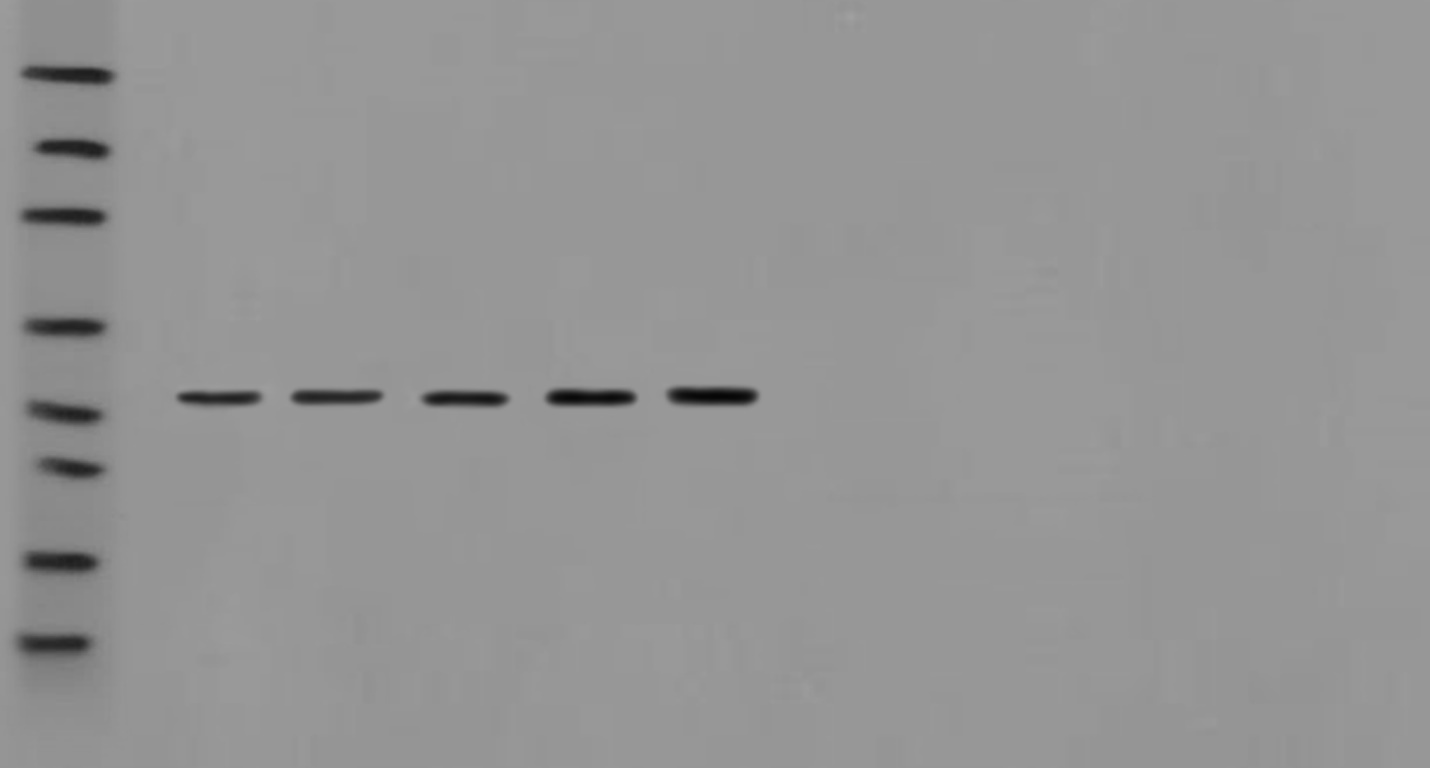


**P21**


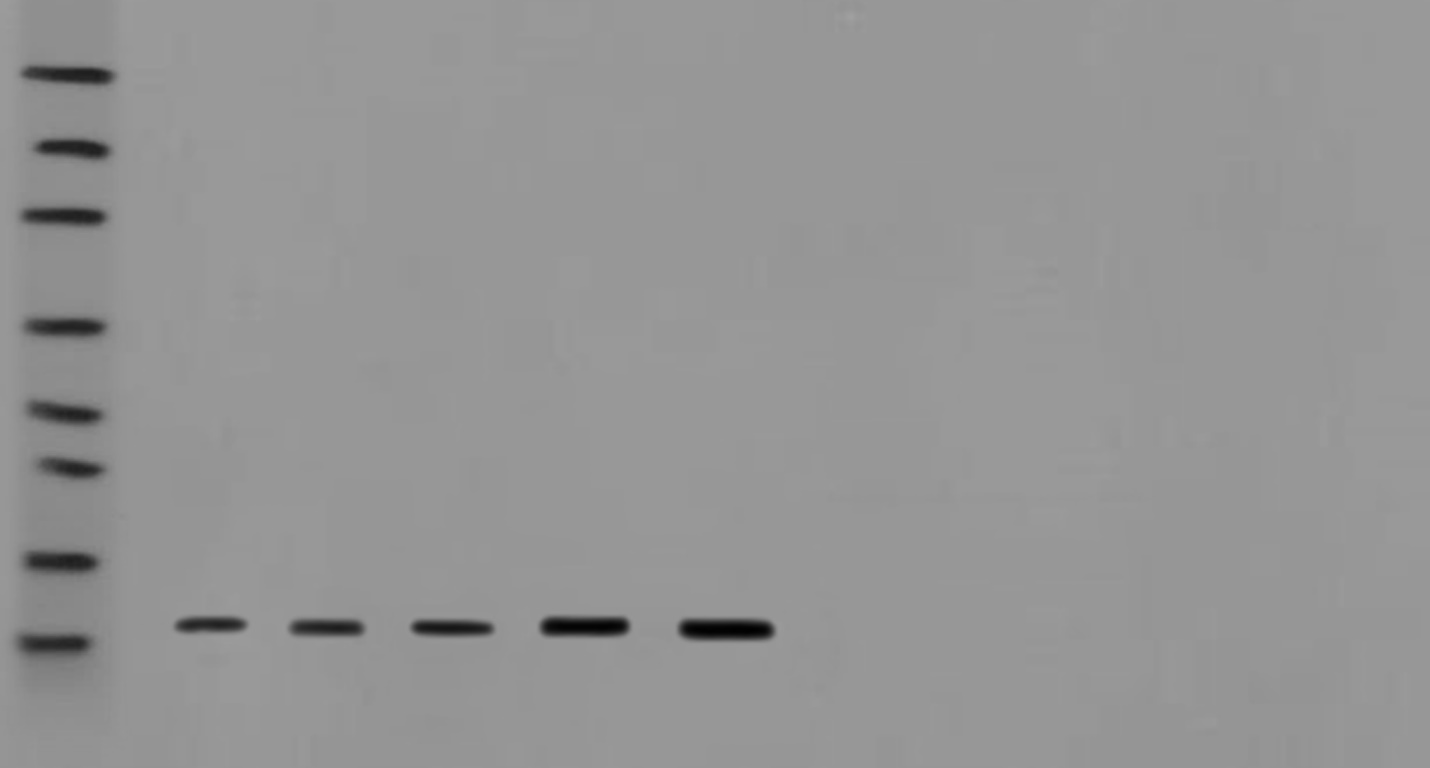


**GAPDH**


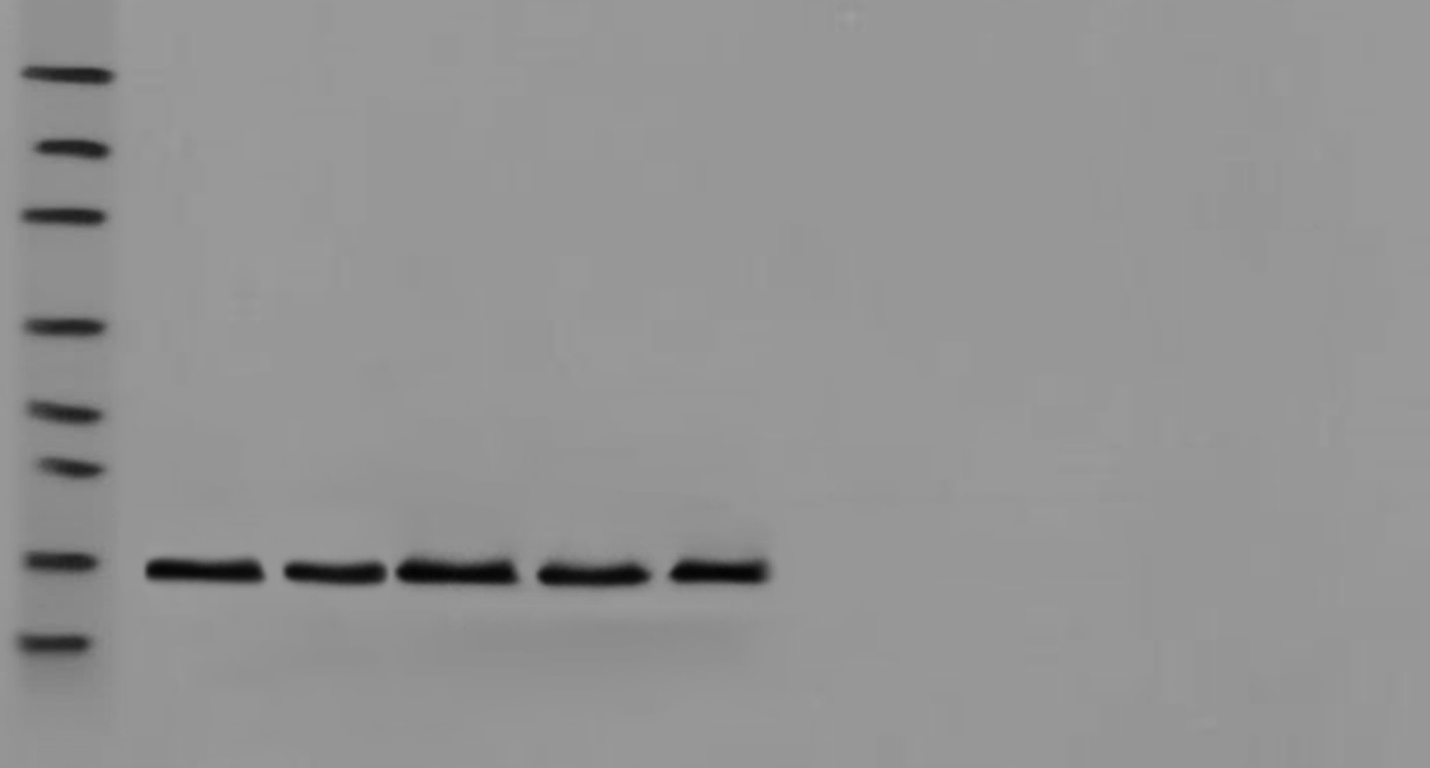

Supplement: Multimedia component 1 [file mmc1.docx]
